# Biomechanical Comparison of WE43-Based Magnesium vs. Titanium Miniplates in a Mandible Fracture Model in Sheep

**DOI:** 10.3390/ma16010102

**Published:** 2022-12-22

**Authors:** Heilwig Fischer, Oskar Schmidt-Bleek, Vincenzo Orassi, Dag Wulsten, Katharina Schmidt-Bleek, Max Heiland, Claudius Steffen, Carsten Rendenbach

**Affiliations:** 1 Department of Oral and Maxillofacial Surgery, Charité—Universitätsmedizin, Corporate Member of Freie Universität Berlin, Humboldt-Universität zu Berlin and Berlin Institute of Health, Augustenburger Platz 1, 13353 Berlin, Germany; 2 Center for Musculoskeletal Surgery, Charité—Universitätsmedizin, Corporate Member of Freie Universität Berlin, Humboldt-Universität zu Berlin and Berlin Institute of Health, Augustenburger Platz 1, 13353 Berlin, Germany; 3 Berlin Institute of Health at Charité-Universitätsmedizin Berlin, Julius Wolff Institute, Augustenburger Platz 1, 13353 Berlin, Germany; 4 Berlin-Brandenburg School for Regenerative Therapies, Augustenburger Platz 1, 13353 Berlin, Germany

**Keywords:** miniplates, WE43, magnesium, osteosynthesis, mandible

## Abstract

In fractures of the mandible, osteosynthesis with titanium plates is considered the gold standard. Titanium is an established and reliable material, its main disadvantages being metal artefacts and the need for removal in case of osteosynthesis complications. Magnesium, as a resorbable material with an elastic modulus close to cortical bone, offers a resorbable alternative osteosynthesis material, yet mechanical studies in mandible fracture fixation are still missing. The hypothesis of this study was that magnesium miniplates show no significant difference in the mechanical integrity provided for fracture fixation in mandible fractures under load-sharing indications. In a non-inferiority test, a continuous load was applied to a sheep mandible fracture model with osteosynthesis using either titanium miniplates of 1.0 mm thickness (Ti1.0), magnesium plates of 1.75 mm (Mg1.75), or magnesium plates of 1.5 mm thickness (Mg1.5). No significant difference (*p* > 0.05) was found in the peak force at failure, stiffness, or force at vertical displacement of 1.0 mm between Mg1.75, Mg1.5, and Ti1.0. This study shows the non-inferiority of WE43 magnesium miniplates compared to the clinical gold standard titanium miniplates.

## 1. Introduction

Fractures of the jaw and midface are caused by sports accidents, acts of brutality, traffic accidents [1], and, mainly in the elderly population, falls [2]. To restore anatomy and function and to allow early mobilization, the gold standard of fracture treatment in adults, especially in dislocated fractures, involves open fracture reduction and internal fixation with miniplates [3]. The most used biomaterial for miniplates is titanium and its alloys [4]. It offers several advantages, including mechanical properties, allowing minimal design and good biocompatibility [4]. The disadvantages include a possible stress-shielding effect, artefacts in postoperative imaging, and the need for metal removal if complications occur or if the patient desires the material to be removed. Complications of permanent implants include the disturbance of skeletal growth, pain, screw loosening, soft tissue complications, and metal-related infections [5]. Magnesium offers a resorbable alternative to established osteosynthesis materials, as reviewed by Wang et al. (2020), with an elastic modulus closer to cortical bone and, thus, a lower risk of stress-shielding [6]. Filli et al. (2015) and Rendenbach et al. (2018) were able to show that magnesium implants produce significantly fewer artefacts in postoperative imaging [7,8]. Additionally, magnesium offers osteostimulative properties, enhancing bone formation [6].

Researchers agree that the control of the hydrogen gas formation during magnesium degradation is the critical step for making magnesium implants suitable for fracture fixation [6,9], reasoning that, if the hydrogen gas formation extends the local resorption capacity, it has the potential to disturb bone healing, a phenomenon described by Witte (2015) [10], among others. To reduce the corrosion rate, and, thereby, the hydrogen gas formation, alloying and surface modifications, such as plasma electrolytic oxidation (PEO), are applied [9,11]. Especially, alloying with passivating elements allows the reduction of the degradation rate to an acceptable speed. In the form of stand-alone screws, the magnesium alloy WE43 is already in human use, and clinical trials have reported comparable results in a direct comparison of the established standard of titanium screws and magnesium screws in foot surgery [12,13] and in the mandible [14]. 

The evaluation methods for new osteosynthesis materials include finite element models, biomechanical tests, and biocompatibility testing in animal models before a material and an implant design can be approved for application in humans. In a finite element analysis, the equivalent mechanical integrity of titanium plates and WE43-based magnesium plates for mandible fracture fixation plates was demonstrated [15]. Miniplate osteosynthesis of the mandible follows the load-sharing principle, allowing a minimal osteosynthesis design operated via an intraoral approach [16]. Two miniplates were used to neutralize both the tensile stress along the alveolar border and the compressive stress along the lower edge [17]. To modify the osteosynthesis bone unit, its different parts can be optimized to increase mechanical integrity. Comparing mandible corpus fractures with miniplate osteosynthesis, increased fixation stability was found when using locking screws in comparison to conventional systems [18]. Increasing the number of screws increased stability, as did the use of y- or double-y-shaped plates [19]. By using a thicker plate at the compression zone, the peak load values showed no significant difference, but the displacement decreased [20].

The biocompatibility of magnesium implants has been shown in both small [21,22] and large animal models [23,24]. WE43-based magnesium miniplates have successfully been tested in non-load-bearing animal models of both the mandible [24,25] and midface [23], but there is still a lack of evidence concerning the biomechanical load-sharing situation of miniplate osteosynthesis of a mandible fracture. To our knowledge, no use of magnesium miniplates in humans has been reported yet. The hypothesis of this study was that WE43-based magnesium miniplates are suitable for load-sharing indications in mandible fractures. To test the biomechanical non-inferiority and the interplay of the bone–material unit of a magnesium miniplate fixation compared to titanium miniplates in mandible fractures, we developed an osteosynthesis model using a sheep mandible to represent a human mandible fracture.

## 2. Materials and Methods

### 2.1. Test Groups

To represent the clinical gold standard, titanium miniplates made of pure titanium were used. Magnesium plates were fabricated from the Mg–Y–RE–Zr alloy WE43MEO (Meotec GmbH, Aachen, Germany); the elemental composition was 1.4–4.2% Y, 2.5–3.5% Nd, <1% (Al, Fe, Cu, Ni, Mn, Zn, Zr), and was balanced with Mg (in wt-%). The alloy was chill-casted and milled to 6-hole locking plates. Plasma electrolytic oxidation with a phosphate-based electrolyte was applied for surface modification of the WE43-based plates.

**Figure 1:**
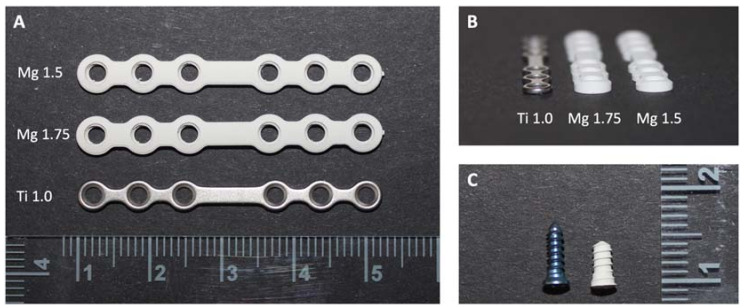
(**A**) Comparison of the different miniplates. All plates had the same number of holes and the same length. Magnesium miniplates were wider, and, within the screw holes, the thread for the locking screws was visible. (**B**) Comparison of the plate profiles. In comparison to the titanium miniplate with a thickness of 1.0 mm, the magnesium miniplates had greater volume. (**C**) Titanium screw (blue, left) 2.0 mm in diameter and 7 mm in length and magnesium screw (white, right) 2.3 mm in diameter and 5 mm in length. The same screw diameter and length were used for both plate thicknesses in magnesium.

The following groups were compared (Figure 1):

– Ti1.0: titanium 1.0 mm (6-hole miniplate titanium) with 2.0 × 7 mm MaxDrive screws;
– Mg1.75: magnesium 1.75 mm (6-hole miniplate WE43-MEO/PEO) with 2.3 × 5 mm MaxDrive locking screws;
– Mg1.5: magnesium 1.5 mm (6-hole miniplate WE43-MEO/PEO) with 2.3 × 5 mm MaxDrive locking screws.

All implants were fabricated by the KLS Martin Group (Gebrüder Martin GmbH & Co. KG, Tuttlingen, Germany). Six samples were tested per group.

### 2.2. Setup for Biomechanical Testing

To ensure a reproducible setup with uniform bones and similar bone quality, sheep heads from approximately one-year-old sheep were bought from a local butcher. The mandibles were stored at −20 °C and defrosted for testing. Mandibles were exposed, and the periosteum was removed to allow a subperiosteal placement of the plates identical to the application of a miniplate in the mandible or midface of humans. In pre-tests, the exact setup, the measurements, and the placement of the plates were defined. For reasons of embedding and as a result of pre-tests, half mandibles separated at the protuberantia mentalis were tested.

**Figure 2:**
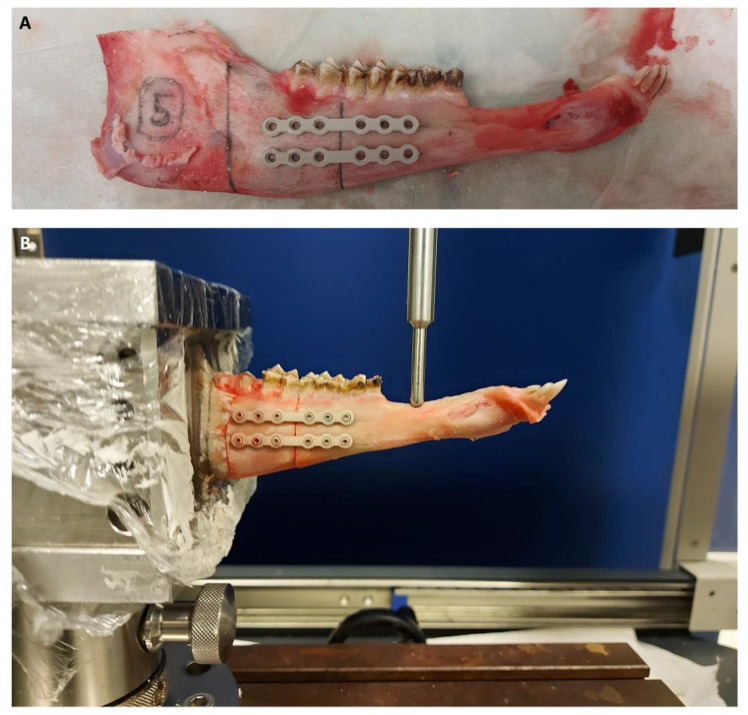
(**A**) Preparation of the half mandible for testing. The periosteum was completely detached, and markings for correct plate placement and the embedding section were applied. A notch for the pole (0.5 cm ∅) applying the force was placed in the diastema with a defined distance of 4.5 cm from the osteotomy to ensure a reproducible application of the force. (**B**) Embedded sample in the materials testing machine (Z010 AllroundLine, ZwickRoell) shortly after the force application started.

A standard protocol for osteotomy and embedding was established. First, a complete osteotomy orthogonal to the row of teeth was placed in front of the first molar using a Piezotome saw (Piezosurgery^®^ flex, Mectron Medical) with a saw blade width of 0.35 mm. Continuous water cooling was applied during the sawing process. To ensure exact repositioning for load-sharing conditions, teeth impeding anatomical reposition were trimmed back slightly at the approximal side using a hand rasp. Originating from the osteotomy, a line was drawn to define the height in the embedding material at a distance of 4 cm. The first plate was placed 0.75 cm from the dental arcade orthogonal to the osteotomy. The second plate was placed at a distance of 0.9 cm parallel to the first one (Figure 2A). To allow the placement inside the embedding jig, the upper part of the ramus mandibulae was detached.

A notch for the pole (0.5 cm ∅) applying the force was placed in the diastema with a defined distance of 4.5 cm from the osteotomy to ensure a reproducible application of the force (Figure 2B).

Before testing, samples were embedded in acrylic casting resin (SCS Beracryl D-28, Suter-Kunststoffe AG). The polymer was mixed with the monomer in a 10:6 volume ratio, according to the fabricant’s instructions. The bones were placed in a self-constructed holding container, fixed on a stand, and adjusted with the help of laser beams. The embedding material was filled in and left to harden for 30 min. The samples were protected from drying out with wet towels during the testing procedure. A total of 18 hemimandibles were tested.

For biomechanical testing, the samples were placed in the materials testing machine (Z010 AllroundLine, ZwickRoell). A two-point bending test of the osteosynthesis bone unit was performed with a continuous vertical movement of 2 mm/s and registration of the applied load. The final endpoint was plate fracture of the first plate, defined as the failure of the fixation method. This was evaluated using the load–displacement graphs and a visual recheck of video sequences taken during the testing procedure. The test setup was developed to mimic the physiological conditions of fracture fixation. Stiffness was determined in the area of 100 N to 125 N. The area of 100 N to 125 N loading was identified as the area where the plates showed linear bending and, therefore, was chosen for the determination of the stiffness of the plates. One sample had to be excluded from the analysis from the titanium group due to an atypical failure mechanism.

### 2.3. Statistical Evaluation

For statistical evaluation and display of the graphs, Graphpad Prism 9 (GraphPad Prism version 9.0.0 for Windows, GraphPad Software, San Diego, CA, USA) was used. Data were compared using the Mann–Whitney U test, while differences in the median were determined using the Hodges–Lehmann difference. Data are displayed as median with 95% CI. Statistical significance was defined as *p* ≤ 0.05.

## 3. Results

**Figure 3:**
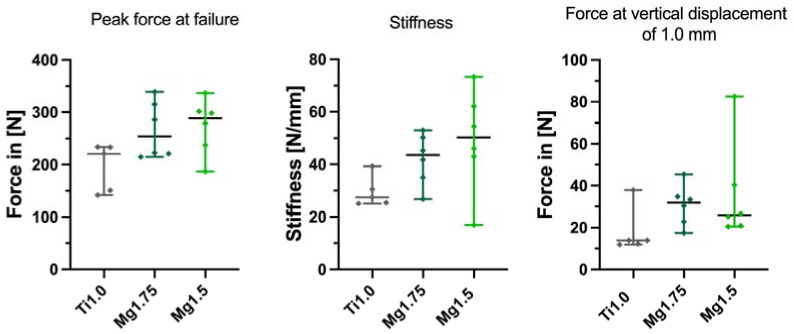
Peak force at failure, stiffness determined in the area of 100 N to 125 N, and force at vertical displacement of 1.0 mm are illustrated. Comparisons of titanium 1.0 mm (Ti1.0), magnesium 1.75 mm (Mg1.75), and magnesium 1.5 mm (Mg1.5) are presented with medians with 95% CI.

The peak forces at failure were higher for both magnesium groups than the titanium group. The median failure of the titanium miniplates (Ti1.0) was observed at 233.3 N, and failure of the magnesium 1.75 miniplates (Mg1.75) was observed at 254.4 N with a Hodges–Lehmann median difference of +39.63 N compared to Ti1.0. The magnesium 1.5 mm miniplates (Mg1.5) failed at a median of 288.6 N with a Hodges–Lehmann median difference of +59.2 compared to Ti1.0 (Figure 3). No significant differences between Ti1.0 and Mg1.75 (*p* = 0.4286) or between Ti1.0 and Mg1.5 (*p* = 0.0823) were found (Figure 3). 

Comparing the stiffness of the bone osteosynthesis unit, the Hodges–Lehmann median difference was −14.03 N/mm between group Ti1.0 (median of 27.46 N/mm) and Mg1.75 (median 43.57 N/mm) and −20.79 N/mm between group Ti1.0 and Mg1.5 (median of 50.28 N/mm). No significant differences were found in the comparison of Ti1.0 and Mg1.75 (*p* = 0.0519) and of Ti1.0 and Mg1.5 (*p* = 0.0823).

The median force at vertical displacement of 1.0 mm showed no significant differences between titanium and magnesium plates. The median force at the vertical displacement of 1.0 mm for Ti1.0 was 13.88 N, for Mg1.75, 31.90 N with a Hodges–Lehmann median difference of −16.48 N (*p* = 0.0823), and, for Mg1.5, 25.92 N with a Hodges–Lehmann median difference of −12.03 N (*p* = 0.0519) (Figure 3).

**Figure 4:**
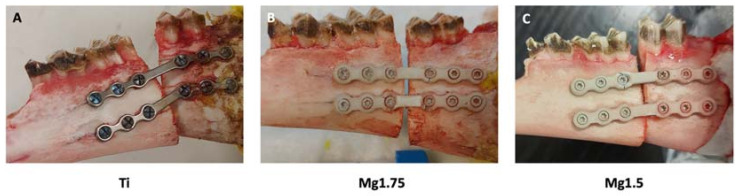
Comparison of plate failure mechanisms. In all cases, a failure of the upper plate occurred. Either the screw proximal from the osteotomy or the screw distal from the osteotomy was identified as the failure location. (**A**) titanium 1.0 mm (Ti) (**B**) magnesium 1.75 mm (Mg1.75) (**C**) magnesium 1.5 mm (Mg1.5).

After osteosynthesis failure, a difference in fracture dislocation was observed between the groups (Figure 4).

**Figure 5:**
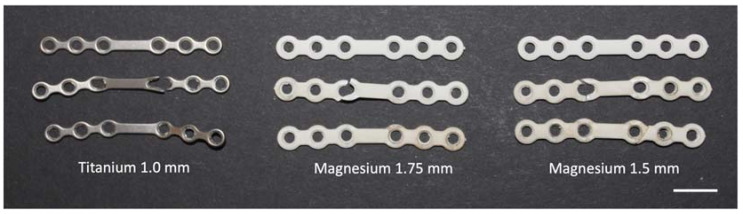
Exemplary plate deformation of the tested plates compared to an original plate (top row). Examples show test samples of the upper plate (middle row) and lower plate (bottom row), with the side facing the mandibular angle facing to the right.

Osteosynthesis failure in group Ti1.0 always occurred at the mesial screw hole adjacent to the osteotomy (Figure 5).

In the Mg1.75 group, three plates failed at the mesial screw hole adjacent to the osteotomy, and three failed at the distal screw hole adjacent to the osteotomy. In Mg1.5, failures were detected on the distal side (four) and the mesial side (two).

## 4. Discussion

This study’s aim was to compare titanium and magnesium miniplates to evaluate magnesium miniplates for fracture fixation in the mandible. To date, there is still a lack of evidence showing the biomechanical non-inferiority of magnesium miniplates in fracture fixation of the lower jaw. In contrast to the established osteosynthesis material titanium, magnesium is a biodegradable osteosynthesis material, and no second operation for metal removal needs to be performed. The rate of manipulate removal shows wide variations between the two materials. In a 10-year retrospective study, O’Connell et al. (2009) described a plate removal rate of 3% [26], while others reported higher removal rates of 6.1% [27] or 22.6% [28], ranging up to 64.1% [5]. The use of resorbable miniplates can both spare the patients risks and pain of the additional operation and ease the economic burden on the health system.

In bone healing, biomechanics can define the success or failure of the outcome. Mechanical stimulation regulates the differentiation of mesenchymal progenitor cells into chondrocytes and osteoblasts [29,30]. The mechanical environment provided by fracture fixation determines the amount of interfragmentary movement. A too rigid fixation or a too flexible fixation can lead to a failure of the bone to restore the bony continuity [31]. In degradable implants, the implant passes with degradation, and, thereby, with the decline of stability, greater and greater strain on the bone results. When degradable implants such as magnesium implants are used, the speed of implant degradation should match the pace of bone healing [32]. If the implant degrades too fast, it might not provide a fracture gap with enough mechanical integrity to restore the bony continuity [33]. In the mandible, the healing of mandible fractures allows patients to take up complete loading again after four weeks [34]. The speed of fracture healing is influenced by the fracture pattern, the patient’s age, possible concomitant diseases, and individual factors [35,36]. 

Magnesium degradation varies decisively between in vitro and in vivo corrosion. Magnesium corrosion in vivo is estimated to be 1–5 times lower than in vitro [37]. In vivo corrosion of WE43 magnesium alloy is estimated to be 0.84 mm year^–1^ [37]. Moreover, Imwinkelried et al. (2013) showed that, 24 weeks after implantation of PEO-coated WE43-based implants, 92 ± 4% of the initial maximum bending force of the implants was maintained [38]. Therefore, this study did not consider magnesium corrosion due to the limited influences of corrosion on the mechanical properties of coated magnesium implants within the first four weeks.

In the literature, sheep hemimandibles are used for biomechanical miniplate testing concerning mandibular angle [39,40] fractures or a sagittal osteotomy of the mandibular ramus [41,42]. Around 25% of all mandibular fractures affect the mandible body [43]. To our knowledge, an osteotomy between the teeth in a sheep mandible to represent a corpus fracture has not yet been described. The use of sheep as a model in bone research offers the main advantage that implants can be tested in their original size because of the similar body weight of sheep compared to humans [44]. The microscopic bone structure of sheep is dominated by a mostly primary bone structure in contrast to the secondary bone structure of humans [44]. Therefore, it can be assumed that there are differences in the biomechanical behavior of different species’ bones. Regarding trabecular parameters, sheep overlap with humans more than pigs [45]. Pigs possess a higher crushing force than humans; like sheep mandibles, their mandible is described as having limited comparability to humans [46]. Despite the differences in bone biology and anatomy, sheep mandibles are considered an established in vivo model in implant research [47]. 

In vivo, magnesium miniplate osteosynthesis with two plates of a complete mandible fracture has already been tested in a minipig model by Imwinkelried et al. (2020). Specifically, magnesium plates with a thickness of 1.45 mm (upper plate) and 1.8 mm (lower plate) were used in combination with 2.7 mm Ø locking screws in a full osteotomy of the mandible behind the most distal tooth. While the lightweight pigs healed (46 and 56 kg, Göttinger minipigs), more giant pigs (70–93 kg, Yucatan minipigs) experienced a failure [48]. This raised the question of whether magnesium miniplates are suitable for mandible osteosynthesis. The reference implants from titanium also failed in the heavier pigs [48]. It can be assumed that the magnesium miniplates might be suitable in cases of mandible fractures where titanium miniplates are sufficient. In contrast to humans, pigs cannot perform partial weight bearing postoperatively and possess a higher chewing force than a human of the same body weight. Those two factors might have additionally contributed to plate breakage in the larger minipigs.

Biomechanical loading in sheep mandibles is not identical to human mandibles. The diastema of a sheep mandible provides the most suitable mechanical loading [49], but placing a double plate osteosynthesis in a sheep diastema results in a placement of two plates being angulated towards each other, and this does not represent the placement of the plates in a similar plane such as the one in a mandibular corpus fracture in humans. Anatomical differences also make the diastema less favorable for a merely biomechanical test setup. Sheep mandibles are approximately twice the length of human mandibles, and placing an osteosynthesis in the diastema thus creates a longer movement arm than that in humans. Additionally, the diastema offers a cross-section different from a human mandible’s corpus. Due to the previously mentioned points, osteosynthesis in the corpus region provides the highest biomechanical comparability to the human situation.

Miniplate osteosynthesis was first described by Michelet in 1973 and modified by Champy et al. in 1978. The technique described miniaturized non-compression plates in combination with monocortical screws 5 to 7 mm in length [16]. The distribution of tensile and compressive stresses during mastication in the mandible allows a minimal osteosynthesis design and an intraoral approach. The mastication process leads to tensile stresses along the alveolar border, whereas compressive stresses dominate along the lower edge. Load-sharing osteosynthesis is only indicated if the fracture pattern allows a reposition where the bone cortices can share the load with the plate, so direct contact between the fragments needs to be achieved [50]. For placing the osteosynthesis plates, Champy defined the ideal line of osteosynthesis. With our sheep model, we reproduced the fixation at the ideal line of osteosynthesis in humans, with one plate close to the alveolar border, neutralizing the tensile stresses, and one neutralization plate to prevent torsion below [17].

We found no significant difference in the peak force at failure between magnesium and titanium miniplates, showing the non-inferiority of magnesium plates compared to titanium plates. The similar peak force at failure in the Mg1.5 and Mg1.75 groups supports the use of thinner plates. Due to the limited number of samples, a high variance occurred, possibly leading to a higher peak force at failure in the Mg1.5 group compared to the Mg1.75 group. Especially in the area of oral- and maxillofacial surgery, as little osteosynthesis material as possible is desirable to improve soft tissue management. Furthermore, with the use of a thinner plate, the amount of hydrogen gas developed in total is reduced. This might reduce the risk of complications. 

Magnesium possesses a lower elastic modulus than titanium [32], which makes it more prone to deformation. Therefore, to compensate for magnesium alloys’ reduced yield strength compared to titanium alloys [32], magnesium plates with a factor in the thickness of 1.5 and 1.75 in comparison to titanium were used in this study. These estimations follow the results published by Imwinkelried et al. (2020), who used magnesium plates ~1.5 times thicker than titanium plates and an adjusted larger screw diameter in an in vivo study [48].

In contrast to other models of titanium miniplate testing in the mandible in the bone, failure occurred in the plates in our model and not at the plate–bone interface [39,51]. In the titanium plates, a more extensive deformation of the screw hole at the point of failure was seen. Additionally, plate deformation was larger in titanium plates. Having identified the point of failure in the upper plate at the screw holes close to the osteotomy, one could propose enforcement of the plates in that specific area or using a thicker upper plate.

Our results showed no significant difference in the stiffness; the scaling factor in comparing the other materials contributed to minimizing the difference in the stiffness between titanium and magnesium. Comparing pure magnesium plates for mandibular angle fixation, a significantly higher stiffness in titanium plates for mandibular angle fixation was shown when comparing plates of the same thickness [39]. In silico results showed that magnesium miniplates ensure sufficient fixation under physiological clenching tasks [15,52]. The occurring dislocation of the mandible parts revealed that material failure is not the only possible failure mechanism of osteosynthesis. Dislocation can negatively affect bone healing and hinder it in extreme cases [53].

The main limitation of this study was the solemnly performed in vitro testing and the non-consideration of the corrosion process of the magnesium implants. Although a sheep model is established for fracture fixation of the mandible, the sheep mandible only offers limited comparability to the lower jaw of a human. Concerning the biomechanical setup, no dynamic testing was conducted. More evidence is needed to provide a deeper insight into the biomechanical behavior, especially under dynamic testing conditions. 

In this study, we presented an easy and reproducible setup for biomechanical plate evaluation in fractures of the mandible body to assess new osteosynthesis materials. In all evaluated plates, a similar failure occurred. In the titanium plates, plastic deformation was visible at the proximal screw hole next to the osteotomy, underlining that the upper plate was stressed in tension in our model, as hypothesized in the model of the ideal line for osteosynthesis. During biomechanical testing with increasing loading, the lower part of the osteotomy ends was compressed and started bearing parts of the force. Despite the compressive stress at the lower borders, no breakage of the bone was visible. Therefore, it can be assumed that the failure of osteosynthesis was always mainly related to the failure of the plates.

## 5. Conclusions

In summary, non-inferiority of both 1.5 mm and 1.75 mm magnesium miniplates compared to 1.0 mm titanium miniplates was demonstrated. With the application of resorbable plates, the patient does not have to undergo the risks and costs of a second operation for implant removal. Biomechanically, this study revealed promising results concerning the use of magnesium miniplates in fracture fixation of human mandibles under load-sharing conditions. 

Future evaluations could include a larger study in a human setup, comparing different fracture scenarios and emphasizing the differences between load-sharing and load-bearing setups in mandible fracture fixation or even reconstructed mandibles. The question remains as to whether magnesium miniplates provide sufficient stability for fracture healing in comminuted mandible fractures or in the atrophic mandible.

## Data Availability

The datasets generated and/or analyzed during the current study are available from the corresponding author upon reasonable request.

## References

[1] Erdmann D., Follmar K.E., DeBruijn M., Bruno A.D., Jung S.-H., Edelman D., Mukundan S., Marcus J.R. (2008). A Retrospective Analysis of Facial Fracture Etiologies. Ann. Plast. Surg..

[2] Sidal T., A Curtis D.A. (2006). Fractures of the mandible in the aging population. Spec. Care Dent..

[3] Kaushik S., Ali I., Dubey M., Bajpai N. (2020). 2 mm Conventional Miniplates with Three-Dimensional Strut Plate in Mandibular Fractures. Ann. Maxillofac. Surg..

[4] Gilardino M.S., Chen E., Bartlett S.P. (2009). Choice of Internal Rigid Fixation Materials in the Treatment of Facial Fractures. Craniomaxillofac. Trauma Reconstr..

[5] Sukegawa S., Masui M., Sukegawa-Takahashi Y., Nakano K., Takabatake K., Kawai H., Nagatsuka H., Furuki Y. (2020). Maxillofacial Trauma Surgery Patients With Titanium Osteosynthesis Miniplates: Remove or Not?. J. Craniofac. Surg..

[6] Wang J., Xu J., Hopkins C., Chow D.H., Qin L. (2020). Biodegradable Magnesium-Based Implants in Orthopedics—A General Review and Perspectives. Adv. Sci..

[7] Filli L., Luechinger R., Frauenfelder T., Beck S., Guggenberger R., Farshad-Amacker N., Andreisek G. (2015). Metal-induced artifacts in computed tomography and magnetic resonance imaging: Comparison of a biodegradable magnesium alloy versus titanium and stainless steel controls. Skelet. Radiol..

[8] Rendenbach C., Schoellchen M., Bueschel J., Gauer T., Sedlacik J., Kutzner D., Vallittu P.K., Heiland M., Smeets R., Fiehler J. (2018). Evaluation and reduction of magnetic resonance imaging artefacts induced by distinct plates for osseous fixation: An *in vitro* study @ 3 T. Dentomaxillofac. Radiol..

[9] Gu X.-N., Li S.-S., Li X.-M., Fan Y.-B. (2014). Magnesium based degradable biomaterials: A review. Front. Mater. Sci..

[10] Witte F. (2015). Reprint of: The history of biodegradable magnesium implants: A review. Acta Biomater..

[11] Chen Y., Dou J., Yu H., Chen C. (2019). Degradable magnesium-based alloys for biomedical applications: The role of critical alloying elements. J. Biomater. Appl..

[12] Atkinson H.D., Khan S., Lashgari Y., Ziegler A. (2019). Hallux valgus correction utilising a modified short scarf osteotomy with a magnesium biodegradable or titanium compression screws—A comparative study of clinical outcomes. BMC Musculoskelet. Disord..

[13] Plaass C., von Falck C., Ettinger S., Sonnow L., Calderone F., Weizbauer A., Reifenrath J., Claassen L., Waizy H., Daniilidis K. (2018). Bioabsorbable magnesium versus standard titanium compression screws for fixation of distal metatarsal osteotomies—3 year results of a randomized clinical trial. J. Orthop. Sci..

[14] Kozakiewicz M., Gabryelczak I. (2022). Bone Union Quality after Fracture Fixation of Mandibular Head with Compression Magnesium Screws. Materials.

[15] Orassi V., Fischer H., Duda G.N., Heiland M., Checa S., Rendenbach C. (2022). In Silico Biomechanical Evaluation of WE43 Magnesium Plates for Mandibular Fracture Fixation. Front. Bioeng. Biotechnol..

[16] Champy M., Loddé J.P., Schmitt R., Jaeger J.H., Muster D. (1978). Mandibular osteosynthesis by miniature screwed plates via a buccal approach. J. Maxillofac. Surg..

[17] AO Foundation ORIF, Two Load Sharing Plates. AO Foundation Website. https://surgeryreference.aofoundation.org/cmf/trauma/mandible/body-simple/orif-two-load-sharing-plates.

[18] Polat M.E., Dayi E. (2019). In Vitro Evaluation of the Effects of Different Fixation Methods on Stabilization of Mandibular Body Fractures. J. Craniofac. Surg..

[19] Ergun S., Ofluoğlu D., Saruhanoğlu A., Karataşli B., Deniz E., Özel S., Tanyeri H. (2014). Comparative evaluation of various miniplate systems for the repair of mandibular corpus fractures. Dent. Mater. J..

[20] Rodrigues D.C., Falci S.G.M., Lauria A., Marchiori É.C., Moreira R.W.F. (2015). Mechanical and photoelastic analysis of four different fixation methods for mandibular body fractures. J. Cranio-Maxillofac. Surg..

[21] Kraus T., Fischerauer S.F., Hänzi A.C., Uggowitzer P.J., Löffler J.F., Weinberg A.M. (2012). Magnesium alloys for temporary implants in osteosynthesis: In vivo studies of their degradation and interaction with bone. Acta Biomater..

[22] Willbold E., Kalla K., Bartsch I., Bobe K., Brauneis M., Remennik S., Shechtman D., Nellesen J., Tillmann W., Vogt C. (2013). Biocompatibility of rapidly solidified magnesium alloy RS66 as a temporary biodegradable metal. Acta Biomater..

[23] Byun S.-H., Lim H.-K., Cheon K.-H., Lee S.-M., Kim H.-E., Lee J.-H. (2020). Biodegradable magnesium alloy (WE43) in bone-fixation plate and screw. J. Biomed. Mater. Res. Part B Appl. Biomater..

[24] Naujokat H., Ruff C.B., Klüter T., Seitz J.-M., Açil Y., Wiltfang J. (2020). Influence of surface modifications on the degradation of standard-sized magnesium plates and healing of mandibular osteotomies in miniature pigs. Int. J. Oral Maxillofac. Surg..

[25] Schaller B., Matthias Burkhard J.P., Chagnon M., Beck S., Imwinkelried T., Assad M. (2018). Fracture Healing and Bone Remodeling With Human Standard-Sized Magnesium Versus Polylactide–Co-Glycolide Plate and Screw Systems Using a Mini-Swine Craniomaxillofacial Osteotomy Fixation Model. J. Oral Maxillofac. Surg..

[26] O’Connell J., Murphy C., Ikeagwuani O., Adley C., Kearns G. (2009). The fate of titanium miniplates and screws used in maxillofacial surgery: A 10 year retrospective study. Int. J. Oral Maxillofac. Surg..

[27] Rallis G., Mourouzis C., Papakosta V., Papanastasiou G., Zachariades N. (2006). Reasons for miniplate removal following maxillofacial trauma: A 4-year study. J. Cranio-Maxillofac. Surg..

[28] Park H.-C., Kim S.-G., Oh J.-S., You J.-S., Kim W.-G. (2016). Mini-plate removal in maxillofacial trauma patients during a five-year retrospective study. J. Korean Assoc. Oral Maxillofac. Surg..

[29] Claes L.E., Heigele C.A. (1999). Magnitudes of local stress and strain along bony surfaces predict the course and type of fracture healing. J. Biomech..

[30] Weinans H., Prendergast P.J. (1996). Tissue adaptation as a dynamical process far from equilibrium. Bone.

[31] Glatt V., Evans C.H., Tetsworth K. (2017). A Concert between Biology and Biomechanics: The Influence of the Mechanical Environment on Bone Healing. Front. Physiol..

[32] Chakraborty Banerjee P., Al-Saadi S., Choudhary L., Harandi S.E., Singh R. (2019). Magnesium Implants: Prospects and Challenges. Materials.

[33] Claes L., Eckert-Hübner K., Augat P. (2002). The effect of mechanical stability on local vascularization and tissue differentiation in callus healing. J. Orthop. Res..

[34] Papel I.D. (2009). Facial Plastic and Reconstructive Surgery.

[35] Einhorn T.A., Gerstenfeld L.C. (2015). Fracture healing: Mechanisms and interventions. Nat. Rev. Rheumatol..

[36] Sheen J.R., Garla V.V. (2021). Fracture Healing Overview. StatPearls.

[37] Sanchez A.H.M., Luthringer B.J.C., Feyerabend F., Willumeit R. (2015). Mg and Mg alloys: How comparable are in vitro and in vivo corrosion rates? A review. Acta Biomater..

[38] Imwinkelried T., Beck S., Iizuka T., Schaller B. (2013). Effect of a plasmaelectrolytic coating on the strength retention of in vivo and in vitro degraded magnesium implants. Acta Biomater..

[39] Abd Al Razik Mohammed A. (2021). Biomechanical evaluation of magnesium plates for management of mandibular angle fracture. Br. J. Oral Maxillofac. Surg..

[40] Cankaya A.B., Kasapoglu M.B., Erdem M.A., Kasapoglu C. (2018). Effects of polymethylmethacrylate on the stability of screw fixation in mandibular angle fractures: A study on sheep mandibles. Int. J. Med. Sci..

[41] dos Santos Trento G., Pires F.A., dos Santos F.A., da Costa D.J., Rebellato N.L.B., Klüppel L.E. (2018). Comparison of the Stability of Mandibular Sagittal Osteotomy Fixation between Two Types of Titanium Miniplates: A Biomechanical Study in Sheep Mandibles. Craniomaxillofac. Trauma Reconstr..

[42] de Olivera L.B., Sant’ana E., Manzato A.J., Guerra F.L.B., Arnett G.W. (2012). Biomechanical in vitro evaluation of three stable internal fixation techniques used in sagittal osteotomy of the mandibular ramus: A study in sheep mandibles. J. Appl. Oral Sci..

[43] Perry M., Holmes S., Perry M., Holmes S. (2014). Mandibular Fractures. Atlas of Operative Maxillofacial Trauma Surgery: Primary Repair of Facial Injuries.

[44] Pearce A.I., Richards R.G., Milz S., Schneider E., Pearce S.G. (2007). Animal models for implant biomaterial research in bone: A review. Eur. Cells Mater..

[45] Watson P.J., Fitton L.C., Meloro C., Fagan M.J., Gröning F. (2018). Mechanical adaptation of trabecular bone morphology in the mammalian mandible. Sci. Rep..

[46] Corte G. (2020). Comparative Cephalometric Studies of the Mandible in Growing Göttingen Minipigs using 3D Computed Tomography: Refining Experimental Dental and Orofacial Research. Ph.D. Thesis.

[47] Ribitsch I., Baptista P.M., Lange-Consiglio A., Melotti L., Patruno M., Jenner F., Schnabl-Feichter E., Dutton L.C., Connolly D.J., van Steenbeek F.G. (2020). Large Animal Models in Regenerative Medicine and Tissue Engineering: To Do or Not to Do. Front. Bioeng. Biotechnol..

[48] Imwinkelried T., Beck S., Schaller B. (2019). Pre-clinical testing of human size magnesium implants in miniature pigs: Implant degradation and bone fracture healing at multiple implantation sites. Mater. Sci. Eng. C.

[49] Orassi V., Duda G.N., Heiland M., Fischer H., Rendenbach C., Checa S. (2021). Biomechanical Assessment of the Validity of Sheep as a Preclinical Model for Testing Mandibular Fracture Fixation Devices. Front. Bioeng. Biotechnol..

[50] AO Foundation Body, Simple. AO Foundation Website. https://surgeryreference.aofoundation.org/cmf/trauma/mandible/body-simple/definition.

[51] Burns B., Fields J.-M., Farinas A., Pollins A., Perdikis G., Thayer W. (2020). Comparing maximal forces in resorbable poly-L-lactic acid and titanium plates for mandibular fracture fixation. Heliyon.

[52] Prasadh S., Krishnan A.V., Lim C.Y.H., Gupta M., Wong R. (2022). Titanium versus magnesium plates for unilateral mandibular angle fracture fixation: Biomechanical evaluation using 3-dimensional finite element analysis. J. Mater. Res. Technol..

[53] Huh J.-Y., Choi B.-H., Kim B.-Y., Lee S.-H., Zhu S.-J., Jung J.-H. (2005). Critical size defect in the canine mandible. Oral Surg. Oral Med. Oral Pathol. Oral Radiol. Endodontology.

